# Developing a social mobilisation intervention for salt reduction: participatory action research in Bombali district, Sierra Leone

**DOI:** 10.1186/s12889-023-16693-6

**Published:** 2023-09-12

**Authors:** Kiran Cheedella, Peter Conteh, Guanyang Zou, John Walley, Ajaratu Kamara, Haja Wurie, Sophie Witter

**Affiliations:** 1 NIHR Research Unit On Health in Situations of Fragility, Institute for Global Health and Development, Queen Margaret University, Edinburgh, UK; 2 NIHR Research Unit On Health in Situations of Fragility and College of Medicine and Allied Health Sciences, University of Sierra Leone, Freetown, Sierra Leone; 3 School of Public Health and Management, Guangzhou University of Chinese Medicine, Guangzhou, China; 4 Nuffield Centre for International Health and Development, Leeds Institute of Health Sciences, University of Leeds, Leeds, UK

**Keywords:** Social mobilisation, Participatory action research, Hypertension, Non-communicable diseases, Salt-reduction, Sierra leone

## Abstract

**Background:**

High salt intake is a major risk factor for hypertension, which in turn contributes to cardiovascular diseases, the major cause of death from non communicable diseases (NCDs). Research is limited on social mobilisation interventions to tackle NCDs, including in fragile health settings such as Sierra Leone.

**Methods:**

Participatory action research methods were used to develop a social mobilisation intervention for salt reduction in Bombali District, Sierra Leone. A team of 20 local stakeholders were recruited to develop and deliver the intervention. Stakeholder workshop reports and interviews were used to record outcomes, enablers, and barriers to the intervention. Focus group discussions were used to observe knowledge, attitudes, and behaviours of community members pre- and post- the intervention.

**Results:**

Stakeholders showed enthusiasm and were well engaged in the social mobilisation process around salt reduction. They developed radio jingles, radio show talks, organised community awareness raising meetings, school sensitisation outreaches, and door to door engagements. Stakeholders reported benefiting personally through developing their own skills and confidence in communication and felt positive about their role in educating their community. The interventions led to reported increased awareness of risks of high salt intake and NCDs, resulting in a reduction of salt use in the community, leading to perceived health gains. However, salt reduction was also met with some resistance due to social factors. Local community structures were also reactivated to work on the interventions and connect the community to the local health facility, which saw an increase in patients having their blood pressure checked. The comparison villages also experienced an increase in awareness and perceived reductions in salt intake behaviours. This was as messages had cascaded via the radio and initial focus group discussions. The social mobilisation stakeholders also agreed on future activities that could continue at no or low cost.

**Conclusion:**

Social mobilisation interventions can provide low-cost strategies to tackle NCDs in fragile settings such as Sierra Leone through the utilisation of community structures. However, more research is required to ascertain the key enablers for replicability and if such successes can be sustained over a longer follow up period.

**Supplementary Information:**

The online version contains supplementary material available at 10.1186/s12889-023-16693-6.

## Background

Non communicable diseases (NCDs), with hypertension as a major risk factor, kills 41 million people every year, equivalent to 71% of all deaths globally [1]. This figure is projected to increase significantly. The burden of NCDs is highest in low and middle income countries (LMICs) where 77% of global NCD deaths occurred [1].

NCDs in Sierra Leone—a fragile and post-conflict country—are on the rise. The Sierra Leonean Ministry of Health and Sanitation (MoHS) developed a National NCD Policy and a National NCD Strategic Plan (2013–2017). A reviewed version was launched in 2020 together with the Sierra Leone Non Communicable Disease and Injury Poverty Commission report. It aimed to address the growing NCD burden, noting limited financial resources to support detection, monitoring, management and control of NCDs. However, there is little data on interventions targeting prevention and management of NCDs in Sierra Leone. A recent review of NCDs in Sierra Leone identified key barriers to tackling NCDs including inadequate health care professionals and financial resources, as well as low levels of knowledge and awareness of NCDs in general [2]. The existing health care system in Sierra Leone is in a rebuilding phase and needs to better address NCDs in the midst of competing priorities, including through tools for management at primary level [3] and better understanding of community perceptions and behaviours in relation to NCDs [4, 5] to inform prevention.

In a recent survey 29% and 31% of male and female adults had raised blood pressure in Sierra Leone, while the percentage of deaths attributable to cardiovascular diseases (CVDs) was 9% (WHO, 2017). Raised blood pressure is a major risk factor for CVD, the leading cause of death in low- and middle-income countries (LMICs). A recent study concluded that CVD risk factors are prevalent and access to care is low [6]. Another study conducted in one of the districts in Sierra Leone, concluded that dietary risk factors for CVD are highly prevalent, particularly among urban residents, and emphasised the need for the development of context specific policies that considers modifiable risk factors for CVD in the context of urbanisation [7].

Excessive salt consumption is not only known to contribute to raised blood pressure but also increases the risk of CVD events and kidney disease [8]. The World Health Organisation (WHO) has recommended reducing salt consumption by 30% by 2025, with a long-term target of less than 5 g/day [9]. While some high-income countries such as Finland and the UK have achieved reduction in average daily salt consumption, accompanied by decrease in blood pressure and CVD mortality of the population, this is challenging in developing countries [10]. Reducing intake of salt, which is usually added by households during cooking or for food preservation, is challenging as changing personal dietary behaviour is difficult [10]. However, one recent study in China has demonstrated the effect of salt substitute on reducing the rates of stroke, major cardiovascular events, and death from any cause among persons who had a history of stroke or were 60 years of age or older and had high blood pressure [11].

Interventions to tackle NCDs in fragile health settings have been limited, as often competing priorities have placed NCDs at the bottom of the list and international support for NCDs has also been lacking [12]. Evidence has shown that health system and community approach is needed to address the barriers to prevention and management of NCDs in LMICs. This study describes a participatory action research project in Bombali district, Sierra Leone, using a social mobilisation approach focused on reduction of salt intake, a major risk factor for CVDs. It considers the potential of social mobilisation to engage communities in Sierra Leone, to define any context specific challenges and co-create sustainable solutions/interventions, with a view to drawing wider lessons for social mobilisation approaches from this pilot study.

Social mobilisation involves engaging community, civil society, traditional and opinion leaders in a common health issue. In addition, government structures (local and national), NGOs and professional groups are involved [13]. Social mobilisation is closely associated with community interventions to engage individuals in key health issues [14–16]. Solutions to these issues are then developed through partnerships, often involving community awareness campaigns and media such as local radio. In social mobilisation, there is a strong emphasis on a community creating their own responses to issues rather than having solutions imposed upon them. This approach is in line with participatory approaches to health issues [17, 18] as well as participatory research [19], as it supports community ownership and leadership. Recent research stresses the importance of developing communication strategies for social mobilisation [20] and building trust and legitimacy to engage communities [21, 22]. The advantages of social mobilisation have included the improvement of the sustainability of programmes (if undertaken using established structures) [23].

Whilst there is a large amount of research on how social mobilisation can address communicable diseases—for example HIV/AIDS [8, 24–26], Ebola [27, 28] and yellow fever [29], there has been little research to date considering how it can address NCDs. Among the limited research, social mobilisation has been used to encourage physical activity in Colombia [30], support to health promotion on reducing NCD risk factors in three Asian and Central American contexts [31] and involving communities in the development of responses to address alcohol and other drug abuse in South Africa [32]. Research is especially lacking on social mobilisation to tackle NCDs in fragile health systems, where there are heightened health needs and limited, often fragmented health provision.

## Methods

This pilot was rooted in a participatory action research (PAR) paradigm [32, 33]. PAR recognises that people’s opportunities for being healthy are affected by social structures and systems. PAR is not therefore focused not just on goals but also on means and emphasises dynamism and interaction, as compared to conventional intervention approaches, which focus on linear causal assumptions. It was therefore adopted as suitable to this social mobilisation pilot, in promoting contextualised documenting of process, participation and iterative learning to identify and share practical experiences [32, 33].

### Setting

The study was conducted in Bombali district, Sierra Leone, building on earlier work to improve primary care management of the most common NCDs (hypertension and diabetes) [3]. Located in the Northern Province and bordering the Republic of Guinea to the north, Bombali District is the second largest district (7,985 km2) in Sierra Leone, with a population of 606,183 in 2015, the majority of whom are Muslim, with a large Christian minority. Bombali district includes 14 chiefdoms, 17 Community health centres (CHCs), 36 maternal and child health posts (MCHPs), 51 community health posts (CHPs) at the primary care level, 3 hospitals, 2 private clinics and 1 district health management teams (DHMT). Livelihoods involve farming staple crops such as rice and cassava, and cash crops such as groundnuts and peppers [34]. The Multidimensional Poverty Index for Bombali is 70%, with the leading indicators being access to electricity, cooking fuel and sanitation [35].

### Social mobilisation process

The pilot aimed to develop and implement a community-based social mobilisation intervention to improve awareness, knowledge, attitudes and behaviour regarding salt intake. This took place among community residents in two rural villages Binkolo and Maforay which are based in the Safroko Limba chiefdom in Bombali district. Binkolo and Maforay have a population of approximately 2600 and 800 people respectively. Both villages are served by Binkolo Community Health Centre (CHC). The CHC, being well engaged with its community members and local stakeholders, facilitated the recruitment of social mobilisation team. The social mobilisation team included stakeholders from the Village Development Committee (VDC) and CHC Facility Management committee (FMC), as well as the district Social Mobilisation lead and the Community Health Officer (CHO) from the CHC and other stakeholders such as teachers and pastors. Criteria for selecting stakeholders included an enthusiasm to be part of the team, a high level of social capital and influence in the community and whose involvement would increase the chances of activities being embedded in existing local structures. The team consisted of 20 participants, of which fifteen participants were men and five women, ranging from 18 years old to 60 years. The disparity in men and women amongst the participants was reflective of the gender imbalance amongst the roles in local structures.

The team were invited to attend four participatory action research workshops to understand the problems of salt intake and CVD in their community, develop an intervention, deliver it and review its outcomes and plan for ongoing activities (see Table 1). The meetings were held in the Krio language with some content in English, in sessions lasting for 4 h each. Refreshments were provided and transport costs of participants were reimbursed. One aim of the social mobilisation process was to improve stakeholders’ knowledge and skills in social mobilisation, CVD and salt reduction. As the workshops progressed and familiarity with the process grew, participants were invited to adopt more active roles (See Table 1). Facilitation was provided by the district social mobilisation lead, along with a local coordinator appointed by the research team. Technical support for messaging and approaches was provided remotely by the international partners.

**Table 1:** Agenda of the PAR workshops

Workshop number	Main aims and activities
1: Introduction to hypertension and social mobilisation	• Discuss hypertension, stroke and COVID-19 with stakeholders • Brainstorm and select activities with stakeholders in relation to salt and other risk factor reduction • Develop a shared understanding of salt and hypertension
2: Co-production of social mobilisation materials and planning for action	• Co-produce materials with the stakeholders e.g. posters, radio messaging and relevant training materials • Work with stakeholders to agree roles and responsibilities in social mobilisation efforts • Check with stakeholders what they want to learn from the project and how they will monitor it
3: Review successes and challenges to date and moving forwards	• Review plans for distribution of messages through stakeholders • Finalise materials, if not done yet • Follow up on successes and challenges of the social mobilisation effort to date
4: Dissemination of findings from research and next steps	• Review of achievements • Plans for moving forward and continuing efforts

Intervention activities were co-developed during the workshops and started to be implemented in the two pilot villages in the Binkolo CHC catchment area after the first workshop between August 2020 and February 2021. We selected another two villages as comparison sites, with similar populations approximately 14 miles apart from the intervention villages for comparison purposes.

The final workshop brought together forty stakeholders (18 female, 24 male), including the team of twenty social mobilisation participants and twenty community representatives from the two villages where activities were conducted. Participants had the opportunity here to reflect on the findings of the interviews and plan future activities.

### Assessment data

Notes were taken during each of the workshops. As well as an attendance register, the process of each workshop was recorded along with relevant issues discussed during each session with agreed outcomes. Reports for each workshop were then generated and submitted to the research team. Qualitative data were also collected to supplement this source. In each village, we conducted focus group discussions (FGDs) of rural residents to gather their views on the content of the materials, their method of delivery and acceptability. The focus groups also aimed to understand participants’ knowledge, attitudes, and behaviours (in relation to the topic of salt intake) before and after the pilot. This was done using a semi-structured topic guide developed by the authors (Supplementary file 1). In total, there were 16 FGDs (8 for baseline and 8 for endpoint). In each village 2 groups (1 female and 1 male) of 8 participants were recruited by the CHO based at the area’s CHC (Table 2) through convenience sampling. For the baseline FGDs in the intervention and comparison sites, the respective CHO recruited participants they had met at community meetings and who appeared active and influential in their villages. Some of the FGD participants from the intervention villages were also stakeholders who went on to be recruited to join the social mobilisation team. The endpoint FGDs in the intervention villages included rural residents who had attended one of the social mobilisation intervention activities, and one or two of those that delivered the intervention. In the comparison villages, the respective CHO again selected villagers he had met at community meetings. All FGD’s were carried out at the respective CHC in both intervention and comparison villages, with only the interviewers and interviewees present. None of the participants refused to be part of the study.

**Table 2:** Samples for FGDs

	Intervention area (2 villages)	Comparison area (2 villages)	Participant numbers
Baseline	4 groups	4 groups	64 (32 males and 32 females)
5-month follow up	4 groups	4 groups	64(32 males and 32 females)

We also conducted five individual interviews with stakeholders from the social mobilisation team using a semi-structured interview guide (Supplementary file 2). Participants were purposively selected, based on those that were most active within the team and in their communities. This included a teacher, two chiefdom heads, a chairman of the VDC and a pastor in the intervention chiefdom. These interviews helped us to understand their experiences of using the materials and delivering the intervention. These interviews were done simultaneously with the FGDs. Each interview ranged from 25 to 30 min each. The interviews were also carried out at the CHC. The sample size of the FGD and individual interviews was pre-determined with the aim of achieving data saturation.

The research team included seven researchers trained in social science, public health and development (4 males and 3 females; three were local researchers; three received a PhD in the UK). A local female research assistant trained in public health and nursing conducted the FGDs and the semi-structured interviews, and there was no prior relationship established to study commencement. The local co-ordinator, with prior experience as a research assistant and trained in development also supported the local researcher in the interviews by means of clarifying questions and answers. Both were involved in the development of the topic guide with the wider research team. Participants were told the research was being carried out to understand salt intake in communities and how social mobilisation can reduce salt intake and reduce high blood pressure and stroke. The participants knew the interviewer was local and worked alongside the College of Medicine and Applied Health Sciences Sierra Leone. All interviews were done in Krio, which is the common local language. All interviews and FGD’s were audio-recorded. The interviews and focus group discussion were mainly conducted in one of the local languages – thus there was a need for translation into English, before transcription. Some interviews were conducted in English and therefore only had to be transcribed. This exercise was carried out by members of the research team and supported by local transcribers who were fluent in the local language in question. Random transcripts were checked for accuracy on transcription or transcription and translation. Transcripts were not returned to participants for comment.

To ensure research ethics in our study, we obtained ethical approval from the institutional review boards (locally and internationally). All the study participants, including those attending the workshops, were reassured of the confidentiality of the research and the anonymisation of their identity. We provided participants with a clear explanation and information sheet of the study's purpose and procedures. The researcher explained the content of the information sheet and obtained consent, and in some cases, we relayed the information in the local language in which the participants were fluent. We gave participants the opportunity to ask questions or seek further clarification before signing or thumbprinting the consent form. We assigned unique identifiers to protect personal information and stored data securely. We followed ethical principles of respect for autonomy, beneficence, non-maleficence, and justice. By taking these steps, we protected participants' rights and well-being and maintained the integrity of our research findings.

### Data analysis

Thematic approach was used to analyse the data. All three sources – workshop reports, interviews and FGDs – were analysed inductively and deductively, while themes were combined if they overlapped. Data were coded by two researchers (See Supplementary file 3 for examples of coding structure). We also piloted a coding frame before applying it to all interviews in each wave of data collection. The narrative data generated from the workshops and interviews mainly related to the process of social mobilisation and outputs generated by community stakeholders, feasibility of the intervention alongside barriers and enablers. The FGD data focused more on community perceptions of NCDs and the effectiveness of the social mobilisation processes and perceived impact on the community.

## Results

The results are presented following the stages of the action research, starting with seeking a shared understanding of the problem and strategies to tackle it, followed by generating action plans, implementing them and reviewing their effectiveness.

### Stage 1: Generating a shared understanding of the problem and possible strategies to address it

#### Sharing principles and understanding of the problem

The first workshop in September 2020 used presentations and group discussion to discuss the challenge of hypertension and how it could contribute to vulnerability to COVID-19. Principles of social mobilisation were also shared, including communicating with appropriate language, building confidence and trust, good listening skills, respect for culture and diversity, active engagement and participation by all. The aim was to build skills with utility well beyond this specific intervention (workshop 1 report).

#### Identifying actors and structures

During this workshop, team members identified community structures that could support their work, and discussed how to engage those structures, potential strategies for social mobilisation, and community entry points in social mobilisation. An initial list of activities to be carried out was drawn up, with an indication of roles and resources needed (Table 3).

**Table 3:** Community structures to support the social mobilisation work

Structure	Roles
Facility Management Committee (FMC)	Supports the facility in coordinating and monitoring all services including drugs and attitude of health workers towards patients; supports campaigns and outreaches; coordinates with the DHMT in cases of staff transfers or staff misconduct
Village Development Committee (VDC)	Supports community developments i.e. shelter for newly transferred staff where there are is inadequate space, land for construction if there is new construction project, settle disputes between the community and the facility. In both the VDC and the FMC members are decision and opinion leaders within the community and the facility
School clubs	Train pupils to support in carrying out advocacy messages on different areas i.e. reduction of salt intake, malaria campaign, etc
Religious organisations	Advocate to their congregation; because of the respect society accords them, their messages are not neglected
The media	Information dissemination. whichever media used, e.g. local radio, the objective is to pass information easily and quickly to a large audience
The District Health Management Team	The represent Ministry of Health and Sanitation at the district level, coordinating and supervising all health facilities and outreach services in the district

The stakeholders agreed that the VDC and FMC should be involved, as they were set up specifically to liaise between services and communities. However, the FMC was reported to be dormant in the intervention side, only active when there is an issue between the community and the facility. Accordingly, the team went on to nominate a wider group of 20 stakeholders in total. This included Pastors and Imams, town chiefs, a Mammy Queen (woman leader who has the responsibility to coordinate issues relating to women and their welfare), youth leader, teachers, traders, and two pupils from the senior secondary level as key stakeholders.

#### Community perceptions of NCDs and salt

Understanding of NCDs within the communities was explored in the baseline FGDs. An NCD was defined in lay terms as a disease that is not transferred and participants were then asked to name one. Close to 40% of the FGD members could not name an NCD, inferring limited understanding. High blood pressure was mentioned by at least one participant in the groups, with diabetes and asthma also stated on occasion. Complications such as heart attacks and stroke were not mentioned. Participants more often stated communicable diseases such as malaria or cholera rather than an NCD. When discussed participants perceived that hypertension is increasing in their communities, and affecting more young people, while it is not easy to identify. They described hypertensive patients as having difficulty in breathing, suffering severe headaches, looking weak, pale, unhealthy and nervous when they walk, being obese, only able to walk a short distance, suffering paralysis of the foot and hand, experiencing spine pain, not able to do their normal activities, and prone to fall down when they walk. While these could relate to symptoms or risk factors for CVD such as stroke or heart attacks, participants did not mention this.

In terms of causes, some participants realised that hypertensive patients should not eat too much salt, including Maggi cubes (a highly salted commercial flavouring commonly used in Sierra Leone). Understanding of the effects of salt was generally limited however and there was denial of it being problematic, as people argued that they had been eating salt and nothing happened to them. People are also attached to its taste enhancing nature, which was also a social issue as food is often shared with neighbours. Women also need to please their husband, as the household head, by ensuring that the meal prepared is palatable to the husband and rest of the family. Poverty was also seen as a barrier to reduction, given lack of alternatives for flavouring food (some reported that even Maggi is not affordable for the poorest). There was also a perception by some that salt provides energy.

I practice I avoid salt, but the condition does not allow us as the poverty is high. There is no alternative except you eat salt. That is why you are well today and after some days you are sick. The condition with us is not good (1^st^ FGD. female).

FGD members talked about several sources of information on hypertension such as radio, books, trainings and workshops and talks in the mosque, church and health centres.

*Through *the* clinic or the radio, sometimes in public meeting that are called by the medical team but not all are invited (1*.^*st*^* FGD, male)*

### Stage 2: Generating a shared set of actions

#### Planning activities

The second workshop was conducted in November 2020. In this step, the participants drew up a set of activities to raise awareness and change behaviour on salt consumption and developed more detail about how to implement them and where (workshop 2 report). Planned activities included:

- Awareness raising meetings at community level
- Door to door engagements at community level
- School sensitisation meetings
- Radio discussion programmes
- Airing of jingles on radio

For each activity, a timeline and target group were established (for example, how many could be reached for awareness raising sessions and where). The wider group discussed the realism of plans presented, to reach a consensus.

For the radio show and jingles, Radio Mankneh (the local radio station) was chosen because it is a community radio and they broadcast in all local languages, and is widely listened to, along with the Sierra Leone broadcasting cooperation (SLBC) as it is the national radio station and has a wide audience. The team also agreed that the jingle should be done in Limba, the local language in the community, and Krio, which is understood nationwide.

They also planned for school outreach, as pupils can pass messages effectively to their parents; they can also be educated to avoid future problems and are involved in much of the cooking at home.

Door-to-door engagement was planned and although acknowledged to be intensive it was felt likely to be effective as it enables more personal, attentive and informal conversations while allowing for a better understanding of household dynamics. During the weekly market day, the team also planned to share public messages more broadly, using megaphones.

#### Agreeing messages

They came up with many messages and agreed on key ones that they felt were sufficiently clear (Table 4). All participants went through the messages and translated them into their local languages (Krio and Limba). Key messages were printed on the social mobilisation T-shirts, banners, posters and stickers, with images that explain to those who could not read and write. The team also developed a radio jingle, made up of the key agreed messages and voiced over by the team in their local languages. One of the banners was used for the school outreach and the other for the awareness raising meetings, carrying different messages but with one key focus on the cross-cutting message: ‘High salt intake can cause high blood pressure, strokes and heart attack’. The team also consulted with the DHMT social mobilisation focal person on the content of the messages developed in this workshop.

**Table 4:** Social mobilisation key messages developed and used

➢ High salt intake can cause high blood pressure, strokes and heart attacks
➢ Reduce your risk of the silent killer disease, high blood pressure, by lowering your salt to less than 1 teaspoon per day
➢ Visit the nearest heath facility for a free blood pressure test. High blood pressure causes strokes and heart attacks
➢ Husbands: support your wives to cook with less salt
➢ Adapt to eating less salt by slowly reducing the amount you cook with
➢ High salt intake can cause high blood pressure

#### Broadening and embedding action

The commitment of the members was acknowledged in this workshop and plans made for continuing activities after the end of the pilot study, aiming to ensure that messages were also taken up by other DHMT social mobilisation activities. Other structures that could support the social mobilisation within their communities were mapped, such as the community town crier, secret society heads, the football clubs, the traditional healers and the local traders’ union. The team agreed to bring in the newly identified community structures to be part of the social mobilisation as they have substantial followings.

### Stage 3: Review of activities

During the third and fourth workshops, held in December 2020 and March 2021 respectively, the facilitator guided the participants to review their activities and any successes, as well as challenges met. The participants suggested that holding monthly meetings was a challenge as they were from several different communities which made meetings unaffordable and too time consuming. The CHO had been checking blood pressures during community activities he attended, however this was not always possible due to lack of functioning BP machines in his CHC. Table 5 highlights the progress reported against the different engagement strategies.

**Table 5:** Report on social mobilisation activities

Activity	Note of achievements
The radio jingle^a^	This was developed and produced by the social mobilisation team in their local language (Limba). The jingle was aired for almost four months on one of the biggest radio programmes in the North: ‘Good Morning Salone’. It is a two-hour programme that brings out issues around the country from various reporters. The radio has a wide listening population every morning and a repeat broadcast in the evening
Radio discussion programmes	These were conducted in Krio and Limba, which are the commonly spoken languages within those communities. The radio programmes were held on two radio stations, the Sierra Leone Broadcasting Cooperation (SLBC) and Radio Mankneh, which is a local but widely listened to. Feedback gathered during the FGDs in comparison communities found that they also received the information through the radio jingles
Community awareness raising meetings	These activities were carried out in eight communities with a total of 240 participants (130 men and 110 women). This activity was facilitated by the social mobilisation team with technical support from the DHMT, CHO and the local research lead. The messages were communicated in local languages, and with involvement of locally respected leaders such as councillors, religious leaders, teachers, and town chiefs, which was seen as critical to the credibility of the messages Community blood pressure testing was carried out after every awareness rising session, leading to detection of new cases
School outreach sensitisation	A total of 500 school pupils were reached with the social mobilisation messages and materials. During the design of the activity, the stakeholders made a strong argument that to disseminate information quickly, students are best placed to do so. This was demonstrated in this study, as parents and guardians made a number of enquiry calls to stakeholders, triggered by information they had received from their children concerning the use of too much salt during meal preparation
Door to door engagement	These activities were carried out in an informal setting, giving the community the opportunity to ask questions and raise concerns. In total, 118 houses were reached within the two communities (Binkolo and Maforay), covering 834 people

^a^https://www.qmu.ac.uk/research-and-knowledge-exchange/research-centres-institutes-and-knowledge-exchange-centres/institute-for-global-health-and-development/nihr-research-unit-on-health-in-situations-of-fragility-ruhf/

### Stage 4: Evaluating the process and planning for next steps

The final workshop was designed as an opportunity for the social mobilisation team and community stakeholders to review the intervention process, and hear information gathered in the FGDs and interviews. They were given the opportunity to look at the key successes of the project, challenges and generate recommendations and activities going forwards.

#### Relevance of engagement on this issue

Community participants identified that they lacked knowledge of the dangers of high salt consumption and associated some of health outcomes, such as strokes and heart attacks, with witchcraft, requiring interventions by traditional healers (workshop 4 report and FGDs). There was also an understanding that they were continuing patterns of behaviour from previous generations, which must therefore be healthy. Reasons for not seeking formal healthcare included poverty and fear of contracting diseases or diagnosis of a feared disease such as Covid-19 or Ebola.

*We are afraid because of the Covid-19, because maybe if I come to the hospital they will say I have Corona so I prefer to go the traditional way.(2*^*nd*^* FGD, female, comparison site).*

Community participants identified high salt consumption as a feature in their lives (workshop 4), including cooking rice with a lot of salt, and eating salt with cucumber, both raw and cooked cassava, potato, gari (cassava powder), popcorn, roasted meat, steamed fish ‘raw soup’, and in biscuits. They perceived alcohol to be a contributing factor as it reduces their sense of taste.

Hypertension was perceived to be a widespread problem in the non-intervention villages, where community members also reported unwillingness to seek formal healthcare for it.

*Some have been *used* to going the traditional way. As they get sick, that is *where* they go. They will not say let me go to the health centre (2*.^*nd*^* FGD, female, comparison site)*

#### Feedback on social mobilisation approaches

The endpoint FGDs and interviews revealed that the intervention was very well received. There was particular appreciation for the clear explanations and demonstrations of how to use salt moderately, the use of local languages and the variety of fora through which messages were conveyed (at home, in health centres, at schools and churches).

*Our sister the councillor went house to house and directed us how to use the salt. If we're using it, it should be one tea spoon, a small one that we should use …she went house to house and directed us using the paper that was given to her (2*^*nd*^* FGD, women, SM area).*

*She said they were in a training today [at school] and I asked her what kind of training and she said "Mama, they said we should not eat a lot of salt anymore". I said it is left with your *mothers* that cook and she said you should tell the ones in the kitchen that they said they should not put a lot of salt anymore (2*^*nd*^* FGD, women, SM area).*

Those involved in the social mobilisation activities were also positive about the approach and materials.

*The materials were very, very good and the process also was timely, looking at the number of *cases* of hypertension (key informant)*

#### Barriers and facilitators to acceptance

Food is shared in households and it can be challenging to try to change cooking habits of other household members. All household members need to be persuaded – women, who cook, and husbands and children, whose feedback is key to maintaining new habits.

*As for me, in my home, my mother is the one that cooks but when I get home, If I tell her when *she* cooks plasas [sauces], to stop cooking plenty salt, she will say, leave alone. I am not cooking for you, I am cooking for my husband. So for me now, I don't have where I will get food except at home, so I think I will not be able to change (2nd FGD, men, comparison site).*

*What will make me change is that before, when I was using three Maggis, my children will say, *Mother*, this sauce today is good. But right now when I cook, they don’t eat that much. They say that the sauce is not delicious. That is what will make me go back to the way I was cooking before (2*^*nd*^* FGD, female, comparison site).*

Eating is often a social activity, and many community participants highlighted the difficulty of rejecting food or criticising neighbours’ food, though over time this could be managed and even used for sharing the messages.

*That is why I too have cut off eat at other people's houses because it is an embarrassing thing to tell them that "you put too much salt". So the best I can do is just stay away…as you want to go into this topic with people they would "just leave us, how long have our people been eating salt and it has done nothing to them". But now as soon as you start it, they would even be the ones to continue it, telling you that indeed they understand what you want to tell them (2*^*nd*^* FGD, men, SM area).*

Some community members also outlined resistance to change, as food prepared with salt and other condiments is perceived as more edible and also a sense that salt was a natural product that they had always consumed. Lack of experience of symptoms or ill health to date was also cited as a reason not to change behaviour.

*Some people said they will not stop that enjoyment; they said salt is enjoyment. So they will not stop it; they will not listen to anybody; they feel that if salt was a bad thing, God would not have created it in the sea. Even when you talk to them, it is the sea that they will *first* refer to, saying "well before, white people were not bringing salt here but we were getting salt from the sea (2*^*nd*^* FGD, men, SM area).*

Poverty was also cited as an important barrier, limiting people’s ability to choose food or influence its preparation, being dependent on others.

*When someone is poor, unless someone give him food. And if the person giving you the food is used to eating plenty salt, and you say you are not going to eat plenty salt, that person will be on the path to death (2*^*nd*^* FGD, male, comparison site).*

On the positive side, the FGDs suggested that residents are motivated to protect their health, including to provide for their families, and stressed the need to continue repeating messages.

*There is yet no medicine to cure this thing, they will just be managing it until the time comes that you finally die; so for me to avoid that sickness from affecting me, maybe I have children to pay for and that thing will make me not to be able to pay for my children; so to take myself away from that, I have to avoid (2*^*nd*^* FGD, men, SM area).*

#### Growing awareness of risks

In the final workshop, key community members highlighted their lack of awareness of the risks of salt previously or of the high salt content of condiments such as Maggi and committed to cascading messages to their communities (workshop 4 report). This was the first time that they had been informed on this topic. Social mobilisation participants reported that it was an outstanding success that the community people now knew that high salt intake could cause high blood pressure (hypertension), stroke and heart attack (workshop 3 report).

The second-round FGDs indicate continued mixed knowledge of NCDs but a greater recognition of the risks and prevalence of high blood pressure and dispelling some previous beliefs about their causes.

*This man I can remember him, when he came and took our BP here, three fourths of us here. You remember right? Three fourths of the people that were here had hypertension, their pressure was high (2*^*nd*^* FGD, men, SM area).*

*It is *changing* gradually with the people because they too are beginning to become aware that salt is a dangerous thing. Because they were expecting that all these things are because of witches; it is now we are realizing that it is not witches (2*^*nd*^* FGD, men, SM area).*

This was confirmed by the key informants, who reported improved awareness in their communities.

*Presently for now, any house you go, they will tell you that indeed that plenty salt is not good, even the children are preaching this message (key informant)*

While the intervention focused on salt reduction, it also educated on other related dietary risks.

*I didn't know that when you fry fish with palm oil and take that palm oil and put it in your *sauce* and eat it, that palm oil will go and stick together in your system and make the blood not to move the way it should move, and that causes high blood pressure (2*^*nd*^* FGD, men, SM area).*

#### Behaviour change

The facility and community screening had also showed that there was increased awareness of hypertension and now people were willing to go for testing to know their status: the number of patients checked and found to have high blood pressure between October and December 2020 were reported by the CHOs to be far more than the previous records in the same periods in 2018 and 2019 (workshop 3 report).

Community members also reported that they had been motivated to go for blood pressure testing (previously not perceived as necessary or available) and to have changed their eating habits because of the messaging (workshop 4 report).

*People are coming for check-up freely, because during the sensitisation we told them that *high* blood pressure check-up is free, you can go to the health facility and check your pressure (key informant).*

FGDs also reported motivation to stay healthy and reduce use of salt, including of Maggi, which is used as a salt substitute. This was even reported to have resulted in reduced sales in salt in the local market. Some members had been given health messages in health facilities before but had not taken these seriously before the social mobilisation messages reinforced them.

*Here in Binkolo, you no longer meet salt on top of a pot's cover. Because how you know that people are doing this, when they put in the sauce, the little that remains they put on the cover, so that if they taste and it is not enough they can add again. But now that does not happen anymore (2*^*nd*^* FGD, women, SM area).*

The intervention also improved healthcare seeking of the residents, according to the final FGDs.

*That knowledge that we had about tradition means that when someone fell down, that it was because of a devil or a secret society, has changed. So now, rather than going to the man *that* is the traditional healer, they prefer to go to the hospital because that is where they are given the right precaution about the sickness in question (2*^*nd*^* FGD, men, SM area).*

#### Perceived health gains

Community members perceived an improvement in health status resulting from reduced salt consumption, including fewer episodes of people falling down and less need to take blood pressure medication.

*The worse of it all was that when the old man brings medicine for me, it is a lot of salt that he would put in there and I would not sleep for the whole of the night. It wasn't until I came back and they tested me and told me about salt; they said "you should be minimizing your salt intake", that was why I got better. It has been many months since I took pressure medication; as long as I do not eat a lot of salt, I have no problem (2*^*nd*^* FGD, women, SM area).*

#### Capacity building for group members

Although a baseline level of skills and capacity for members of the social mobilisation team was not carried out, there was a self-reported improvement. This included a growth in skills and confidence in communication and addressing groups, as well as positive feelings about their role in educating their communities.

*I was not fortunate to go to school, so I cannot read or write but my role as a TBA has been *helpful* within the community because I have referred many pregnant mothers to the health facility. What inspired me during the training was the model of communication. They have called us for many workshops and trainings but the language of communication was a major barrier. But for this project, the facilitators gave us the opportunity to ask questions and participated well because it is our own local language (Limba). During the school outreach sensitisation, I was given the opportunity to talk to the school pupils and I did it well because it is in our own local language. Since that day I have reached onto many with the messages and the social mobilisation for this project is now part of my daily activity, because I want to save my people with the little knowledge I have (SM participant, workshop 4 report).*

#### Reviving local structures

Another result was the activation of the VDC and FMC, which had previously existed on paper but were not active; they were now functioning and connecting the community and the facility (workshop 3 report).

#### Benefits for comparison sites

Although activities were not conducted in the two comparison villages, FGD participants cascaded information they had learnt in their discussion to other community members. Radio programmes from the intervention had also reached the comparison villages. This led to some positive changes including a perception of reduced salt consumption, which community members believed to have led to a reduction in ill health.

*They said on the radio that eating too much Maggie is not good, especially this white Maggie [monosodium glutamate] …If you want to eat it, you should eat a small amount of it but putting it plenty in a sauce is not good (2*^*nd*^* FGD, female, comparison site).*

*I was able to learn that most of the sickness that attacks us here like high blood pressure have reduced because we have learned that eating plenty salt can lead to getting those diseases. So since that time, in my own home, we have not been eating salt anymore (2*^*nd*^* FGD, female, comparison site).*

#### Planning future activities

The social mobilisation participants identified activities which were low or no cost to continue on a regular basis after the research ended, with the group continuing to meet every month. Each activity had a lead person allocated to it. These included:

1. Door to door engagement by the group, wearing identifiers – i.e., their t-shirts,
2. Market outreach including the weekly ‘Luma’ market days where traders from far and wide (both within and outside the community) bring their goods for sale
3. School outreach to be continued during the morning devotions, to be headed by teachers in their schools.
4. Awareness raising activities in churches and mosques to be carried out by pastors and imams.
5. CHW capacity building with salt reduction messages by the CHO.
6. Health talks on salt intake reduction during the ANC, PNC and immunization clinics by the CHO.
7. Community and facility screening for high blood pressure to be carried out by the CHO.

The FGD participants also made suggestions for future engagements, including continuing the trainings and adding more visual images to materials. Key informants also suggested that drama would be an effective approach.

*More pictures of the ones it has affected because they learn more as they value pictures more *than* anything…. the salt, they should have its picture; the Maggi, they should have its picture and also underneath there, write the dangers involved with it (2*^*nd*^* FGD, men, SM area).*

#### Barriers and enablers for social mobilisers

Practical challenges facing the social mobilisation team included transport to reach villages and funds to support communication for planning.

*They *are* inaccessible so we have to walk for some meters or some miles and when you get there, maybe you will not meet the people there (key informant)*

Key informants also identified practical enablers and positive lessons, including:

- ✔ Having clear messages
- ✔ Using pictures and practical demonstrations
- ✔ Using existing structures such as teachers in schools (school outreach was seen as particularly effective) and pastors in churches
- ✔ Following up – the importance of repeating messages
- ✔ Using the local language- Limba
- ✔ Working with local authorities – the collaboration with the DHMT and the VDC was particularly important
- ✔ The key role of the CHO in the mobilisation activities was also important to link communities to the health services more effectively
- ✔ Working during the dry season so that villages are accessible
- ✔ Having transportation such as a bike and supportive equipment such as blood pressure machines
- ✔ Being well presented, clean and polite

*During that sensitisation we go around with the BP machine and checked them all. Out of the 40, 21 were having high blood pressure. So that made them to believe that if they eat too *much* of salt, they will have stroke, hypertension and heart failure. So they were so surprised, amazing and they accept the fact that they should not eat too much salt… The messages were well received by the community and anywhere we go for sensitisation, we do screening to see the level of high blood pressure in that community (key informant).*

*CHOs were taking good parts because they were also talking to the people, telling them that to do *the* test is free and that people should not be afraid of the hospital. They told people to go to the hospital, go the clinic. If you go to the hospital, you will get good health (key informant).*

## Discussion

This was a modest intervention, with field costs of approximately $17,000, lasting fewer than six months and reaching 2 villages comprising of 3400 people in total, with the radio show potentially reaching out across the whole district with an estimated population of 606,183 in 2015. Promising results from the interventions included a growing awareness amongst the community of risks and causal factors for hypertension, especially the role of salt consumption, as well as the willingness to be tested and to act on the messages of reduced salt consumption. Given the scale of hypertension in Sierra Leone and globally [1], this is an important conclusion. Our evidence is largely qualitative at this stage but indicates the acceptability of the social mobilisation approach adopted, which also generated benefits for the core group of participants, in terms of knowledge and confidence, as well as reviving, at least temporarily, local structures such as the VDCs.

In addition to contributing to change in the two focal communities, our FGDs in two comparison villages which were initially conceived of as independent comparators suggests that salt reduction messages have reached a broader area. This was achieved through the FGDs themselves, the supported radio programmes, health staff and, likely, word of mouth.

Previous studies showed that comprehensive community-based interventions comprising structural change, community mobilisation, health education and social marketing at scale were effective in managing the risk factors of NCDs in developing countries [31, 36]. Our results are consistent with this study and further indicate some of the factors that supported these positive results, including reviving of the community structures, the central role of social mobilisers representing important local groups and constituencies (including the health centre staff), the engagement of locally trusted influencers (such as teachers and religious leaders), and the use of the local languages and visuals which helped to communicate effectively to people lacking English or Krio and some of whom are illiterate. The fact that the messaging targeted different age groups, and especially the young, seems to have been supportive, ensuring that households receive messages from different sources, many of which are persuasive. Some households had previously been advised on hypertension and salt consumption by health staff, but now that they were hearing the same message from other sources closer to their home, they reported now taking them seriously.

This article adds to a very limited literature from LMICs and fragile states which investigates how social mobilisation may support the fight against the growing burden of NCDs. Its findings on low community awareness and the prevalence of traditional beliefs about causal factors supports articles which noted that the key barriers to tackling NCDs include inadequate health care professionals and financial resources, as well as low levels of knowledge and awareness of NCDs in general [2] and that Sierra Leoneans hold entrenched beliefs which imply disease onset to be associated with spiritual causes such as evil spirits or witchcraft [5]. It suggests scope for modification, however, even though the wider literature indicates that modifying personal dietary behaviour is challenging [22]. We note resistance linked to the social nature of food preparation and consumption and therefore the need to consider preferences and status of other household members and neighbours. However, a community approach which targets different genders and age groups appears able to address this, at least in the short term. Women have agency through their role in cooking, men as household heads, the elderly by virtue of status and the young as influencers and consumers. Messaging therefore needs to target all segments to ensure that behaviour change is effective and sustained. Co-creation of these messages can promote community ownership and leadership, which can prevent fragmentation and loss of investment in these efforts. The use of schools as an outreach platform is also potentially of great benefit to support behavioural change at a young age and transitioning into adulthood.

The article contributes to a growing body of evidence on the value of social mobilisation and participatory approaches in which communities create their own responses to issues, rather than having solutions imposed on them [23, 33]. Although the topic in this pilot was not nominated by the community, the strategies to address the issue were developed by the social mobilisation team, comprising a variety of local stakeholders, with external facilitation but without any preconceived approaches. This is likely one of the main factors for its reported success, as local engagement, ownership and tailoring to local perceptions and needs were high.

We need to note some important limitations of this study, including its relatively short duration and small geographical area. This makes it indicative of powerful approaches to tackling NCD risk factors in this and similar settings, rather than conclusive. It is also important to recognise overlapping roles, as is common in participatory approaches: one of the interviewers was also involved in the development of the intervention, as were some of the interviewees, which may have influenced responses. Further research would be needed to quantify the effectiveness of the intervention using similar comparison sites and a before-after design. Strategies should also be developed to scale up and sustain such approaches, also examining their effectiveness in urban areas (it is possible that the rural setting for this work supported its reported effectiveness).

## Conclusion

This social mobilisation intervention was delivered at low-cost, utilising community structures within a fragile health system to tackle the rising burden of NCDs in 2 villages in Sierra Leone. Through community members developing their own interventions and involving all ages and opinion leaders there is potential for knowledge, attitudes, and behaviours to change, with potential for sustainability in relation to development of community capacities and strengthening of local structures. However, more research is required to ascertain the key enablers for replicability and if such successes can be sustained over a longer follow up period.

### Supplementary Information

**Additional file 1. **Interview topic guides for focus group discussion (baseline interviews for both intervention and control groups).

**Additional file 2. **Interview topic guides for Key Stakeholders.

**Additional file 3. **Examples of coding structure for projected titled ‘Developing a social mobilisation intervention for salt reduction: participatory action research in Bombali District, Sierra Leone’.

## Data Availability

The datasets generated and/or analysed during the current study are available from the corresponding author on reasonable request.

## Abbreviations

NCD: Non Communicable Diseases
CVD: Cardiovascular Disease
CHC: Community Health Centre
VDC: Village Development Committee
FMC: Facility Management Committee
FGD: Focus Group Discussion
SM: Social Mobilisation
DHMT: District Health Management Team
WHO: World Health Organisation
