# Supplementary material for: Developing a social mobilisation intervention for salt reduction: participatory action research in Bombali district, Sierra Leone
Source: BMC Public Health. 2023 Sep 12;23:1774. doi: 10.1186/s12889-023-16693-6 (PMC10496325; doi:10.1186/s12889-023-16693-6)
Supplement: Supplementary file 1 — Additional file 1. Interview topic guides for focus group discussion (baseline interviews for both intervention and control groups). [file 12889_2023_16693_MOESM1_ESM.docx]

**Supplementary file 1**

**Interview topic guides for focus group discussion (baseline interviews for both intervention and control groups)**

- Can you introduce yourselves? (start with a quiz/test on NCD knowledge)
- Do you receive any health related intervention in this village in the last 12 months?
- How are your health conditions? (e.g., do you have hypertension or other NCDs)?
- If you have adverse conditions, do you seek care? Why or why not？
- Can you talk about your lifestyles, especially regarding salt intake?
- Do you know about the harm of keeping unhealthy lifestyles, especially regarding using too much salt?
- Do you have any access to educational information related to NCDs? What are the main sources? (e.g., health workers, radio, posters)
- Do you want to change your lifestyles, especially reducing salt intake? Why or why not?
- What would help you to start to make a change? (e.g., health systems, community, social culture etc.)
- If you have started to make a change, what are the barriers and enablers to the change? (e.g., health systems, community, social culture etc.)
- Do you have suggestions for us to provide a feasible and sustainable community social mobilization intervention for reducing salt intake?

**Interview topic guides for focus group discussion for rural residents (follow up interview for intervention group)**

*Please probe for variation throughout*

Can you introduce yourselves? (start with a quiz/test on NCD knowledge) – note similarities and differences):

- can you give an example of a non-transferable disease?

- why do we get high blood pressure?

- what can we do to reduce it?

How are your health conditions? (e.g., do you have hypertension or other NCDs)? If you have adverse conditions, do you seek care? Why or why not？

Have you seen or heard any messages about keeping healthy and preventing problems with high blood pressure?

- What were they? Where did you hear them? Did they prompt to make any changes? If so, which?

Have you been involved in any of the following over the last few times?

1. Radio jingles
2. Door to door awareness raising
3. School sensitization
4. Community meetings
5. Community screening – CHO outreach

For each element above, probe:

- Did you learn anything from it?
- If so, what?
- What changes did you make, if any?
- If not, why not? What influences that? (e.g. gender)

If you have started to make a change, what are the barriers and enablers to change?

- Which components of the intervention are most useful? (see list above)?
- What improvement do you think we should make in terms of materials and intervention delivery?

**Interview topic guides for focus group discussion for rural residents (follow up interview for control group)**

*Ideally the same participants as the baseline; please probe for variation throughout*

Can you introduce yourselves? (start with a quiz/test on NCD knowledge – note similarities and differences).

Can you give an example of a non-transferable disease?

- why do we get high blood pressure?
- what can we do to reduce it?

How are your health conditions? (e.g., do you have hypertension or other NCDs)?

- If you have adverse conditions, do you seek care?
- Why or why not？

Have you seen or heard any messages about keeping healthy and preventing problems with high blood pressure?

- What were they? Where did you hear them? Did they prompt to make any changes? If so, which?

Have you started to make changes about your lifestyles (diet, exercise, drinking and smoking) during the last few months? Why or why not?

If you have started to change your lifestyles, what are the barriers and enablers for you to change?

- How is this different for different community groups?
- What would help you in future to change your lifestyle to be healthy?
