# Supplementary material for: Developing a social mobilisation intervention for salt reduction: participatory action research in Bombali district, Sierra Leone
Source: BMC Public Health. 2023 Sep 12;23:1774. doi: 10.1186/s12889-023-16693-6 (PMC10496325; doi:10.1186/s12889-023-16693-6)
Supplement: Supplementary file 2 — Additional file 2. Interview topic guides for Key Stakeholders. [file 12889_2023_16693_MOESM2_ESM.docx]

**Supplementary file 2**

**Interview topic guides for Key Stakeholders**

Materials and intervention development

What did you think of the process of the intervention and materials development?

- What helped you to work together well with other stakeholders to develop the interventions? Were there any barriers to working together well? Are there any improvements that could be made to the process?
- What did you think to the process of developing the materials? Was it successful, if so why? Were there any challenges? What improvements could be made?
- How many workshops did you attend? Did you meet with stakeholders outside of the main workshops to make plans? Were there any improvements you think that could have been made?

Training

Can you comment on the training you received in the workshops? (approach, process and effect and material improvement)

Feasibility of the intervention

- What is your role in the intervention delivery?
- What do you think of the key interventions? What were the successes? What the challenges? What were the most effective? Are there any improvements that could be made?
- What are the barriers and enablers of delivering this intervention? (e.g., health systems, community and social structures, culture etc.)
- What did you think the community members thought about the interventions and materials? (excited, indifferent, not important to them?) How could they be better engaged?
- How well did community structures like the VDC support the delivery of the intervention, if at all? How could they be better engaged?
- How have government structures like the DHMT or health centre supported the delivery of the interventions, if at all? How could they be better engaged?
- Do you notice whether the residents have started to make changes about their lifestyles especially regarding salt intake since the intervention delivery? If so, what? Why or why not were they able to make changes?
- What lessons do you draw for the most effective way to get households and communities to reduce salt intake in future, and in general to protect their health?
