# Supplementary material for: Developing a social mobilisation intervention for salt reduction: participatory action research in Bombali district, Sierra Leone
Source: BMC Public Health. 2023 Sep 12;23:1774. doi: 10.1186/s12889-023-16693-6 (PMC10496325; doi:10.1186/s12889-023-16693-6)
Supplement: Supplementary file 3 — Additional file 3. Examples of coding structure for projected titled ‘Developing a social mobilisation intervention for salt reduction: participatory action research in Bombali District, Sierra Leone’. [file 12889_2023_16693_MOESM3_ESM.docx]

**Supplementary file 3**

**Examples of coding structure for projected titled ‘Developing a social mobilisation intervention for salt reduction: participatory action research in Bombali District, Sierra Leone’**

Focus Group Discussion (FGD)

- [Baseline study of social mobilization intervention](#_Toc72521465)
- [Perception of non-communicable diseases](#_Toc72521466)
- [Perception of hypertension (such as symptoms and health seeking behavior)](#_Toc72521467)
- [Perceived impact of over-salt intake](#_Toc72521468)
- [Suggestions of education and intervention approaches](#_Toc72521469)
- [Potential barriers of salt reduction](#_Toc72521470)
- [Follow up interview of social mobilisation intervention (intervention group)](#_Toc72521471)
- [Health condition](#_Toc72521472)
- [Perception of symptom and salt to HTN](#_Toc72521473)
- [Content of education](#_Toc72521474)
- [Perception of intervention](#_Toc72521475)
- [Impact of intervention](#_Toc72521476)
- [Motivation and Barriers to change](#_Toc72521477)
- [Suggestion for the intervention](#_Toc72521478)
- Follow up interview of social mobilisation intervention [(control group)](#_Toc72521479)
- [Health condition](#_Toc72521480)
- [Venue of seeking care](#_Toc72521481)
- [Change of lifestyle](#_Toc72521482)
- [Motivation and barriers to change](#_Toc72521483)

K[ey stakeholder interviews](#_Toc72521484)

- [Perspective of participatory workshops (Materials and intervention development)](#_Toc72521485)
- [Experiences of working together with others, including enablers and barriers](#_Toc72521486)
- [Process of developing the materials](#_Toc72521487)
- [Perception of participatory workshops (including benefits of the training, e.g. approach, process and effect and material improvement)](#_Toc72521488)
- [Whether working with others outside the workshop](#_Toc72521489)
- [Feasibility of the intervention](#_Toc72521490)
- [Role of the stakeholders(interviewees)](#_Toc72521491)
- [Perception of the intervention, including successes (and examples on effect of salt reduction) and challenges and suggestions for improvement](#_Toc72521492)
- [Impact on healthcare seeking behaviour](#_Toc72521493)
- [Impact of intervention on community's attitudes and behaviour](#_Toc72521494)
- [Enablers to intervention delivery](#_Toc72521495)
- [Impact of government structure on intervention](#_Toc72521496)
